# Bidirectional Regulation of Opioid and Chemokine Function

**DOI:** 10.3389/fimmu.2020.00094

**Published:** 2020-01-31

**Authors:** Thomas J. Rogers

**Affiliations:** Center for Inflammation, Translational and Clinical Lung Research, Lewis Katz School of Medicine, Temple University, Philadelphia, PA, United States

**Keywords:** GPCR, desensitization, inflammation, pain, migration

## Abstract

The opioid family of GPCRs consists of the classical opioid receptors, designated μ-, κ-, and δ-opioid receptors, and the orphanin-FQ receptor, and these proteins are expressed on both neuronal and hematopoietic cells. A number of laboratories have reported that an important degree of cross-talk can occur between the opioid receptors and the chemokine and chemokine receptor families. As a part of this, the opioid receptors are known to regulate the expression of certain chemokines and chemokine receptors, including those that possess strong pro-inflammatory activity. At the level of receptor function, it is clear that certain members of the chemokine family can mediate cross-desensitization of the opioid receptors. Conversely, the opioid receptors are all able to induce heterologous desensitization of some of the chemokine receptors. Consequently, activation of one or more of the opioid receptors can selectively cross-desensitize chemokine receptors and regulate chemokine function. These cross-talk processes have significant implications for the inflammatory response, since the regulation of both the recruitment of inflammatory cells, as well as the sensation of pain, can be controlled in this way.

## Introduction

Opioid receptors are members of the seven transmembrane G protein-coupled receptor (GPCR) superfamily, and based on amino acid sequence similarity, these receptors are a part of the class A (or rhodopsin-like) GPCR family (1–6). The opioid receptor sub-family is composed of μ-, κ-, and δ-opioid receptors (MOP, KOP, DOP), and the orphanin-FQ receptor, or opioid receptor like-1 (NOP). These four receptors share roughly 70% amino acid sequence homology (7). While opioid receptors are expressed predominantly in the nervous system, it is clear that leukocytes also express the opioid receptors (8–11). Moreover, several functions of cells of the immune system are modulated following the activation of the opioid receptors (12–14).

The chemokine receptors are also members of the class A GPCR family, and are classified into four types based on the terminal cysteine amino acid sequence of the respective agonists (C, CC, CXC, and CX_3_C). The chemokine receptors can play a critical role in a variety of processes, including hematopoiesis, inflammation, resistance to infections, and organogenesis (15, 16). It is clear that chemokine expression is critical for the inflammatory response, and an increase in chemokine expression is associated with a wide range of inflammatory diseases. These proteins are involved in both innate and adaptive immunity, and play important roles in leukocyte recruitment and organ positioning, integrin activation, leukocyte degranulation, angiogenesis, and monocyte surveillance (16, 17).

The opioid receptors regulate inflammatory processes at multiple levels. For example, opioid receptor activation can result in alterations in pro-inflammatory cytokines and chemokine gene expression. This includes MOP-mediated down-regulation of the expression of TNFα (18), and the up-regulation of CCL2 expression (19–21). Opioid receptor activation can also lead to the regulation of chemokine receptor gene expression. For example, the activation of MOP leads to a significant up-regulation of expression of both CCR5 and CXCR4 by both monocytes and T cells (22), while the activation of KOP leads to an inhibition of the expression of CXCR4 (23). The signaling processes which are responsible for the opioid-mediated control of chemokine and chemokine receptor gene expression will be reviewed in this paper.

Opioid receptors may also regulate the functional activity of chemokine receptors through the process of heterologous desensitization. This is a process in which the activation of the opioid receptor initiates a signaling pathway which leads to the inactivation (or desensitization) of an unrelated GPCR in the absence of ligand for the second GPCR (24). There is a clear degree of selectivity in the capacity of GPCRs to carry out cross-desensitization, and certain receptors are highly resistant to this type of regulation. Moreover, certain chemokine receptors can mediate cross-desensitization of the opioid receptors, and lead to a loss of sensitivity to opioid agonist administration. Overall, the cross-talk between GPCRs can have substantial consequences, particularly for the inflammatory response. In this review, the biochemical basis for the bi-directional cross-talk between opioid and chemokine receptors will be described.

## Opioid Receptor Signaling Processes

Both opioid and chemokine receptors are coupled to the αβγ G protein complex, and upon activation the G proteins are uncoupled, releasing the α and βγ chains. The Gα subunits are classified into four groups: Gα_i/o_, Gα_s_, Gα_q_, and Gα_12_. The opioid and chemokine receptors are G_i_/G_o_-coupled receptors, and following receptor activation, these G proteins are then able to initiate a variety of signaling cascades. For example, the released G proteins inhibit adenylyl cyclase, Ca^2+^ channels as well as stimulate K^+^ channels and increase intracellular Ca^2+^ levels (25). The release of Gβγ proteins appears to serve a substantial role in the initiation of a number of signaling pathways, and may be essential for cell migration (26–28). Moreover, the control of cell migration appears to be dependent on Gβγ proteins released from G_i_-coupled, but not G_s_- or G_q_-coupled receptors (26).

The Gβγ-initiated signal transduction pathways can regulate a number of critical cellular functions, including cell growth and differentiation, due in part to the induction of the mitogen-activated protein kinases (MAPK) (29). All of the opioid receptors have been shown to activate the ERK1 and ERK2 MAPK family members, and one or more of the opioid receptors also induce both JNK and p38 MAPK pathways as well (30, 31). The capacity of the opioid receptors to activate the MAPK cascades is particularly important since these pathways play critical roles in the function of inflammatory cells.

The opioid Gβγ subunit also signals to the downstream effector phosphoinositide 3-kinase (PI3K), a signaling pathway that often leads to growth activation (32). In addition, PI3K is often required for the activation of the nuclear factor-κB (NF-κB) signal transduction pathway, an additional critical element involved in pro-inflammatory gene expression (33–36). Both MOP and DOP have been shown to initiate the PI3K pathway to promote cell survival and inflammatory pain (37, 38).

The activation of GPCRs often results in the induction of the NF-κB signaling pathway, as a result of the up-stream signaling cascades including protein kinase A (PKA), PI3K, or PLCβ (39). This includes the formyl peptide receptor, CXCR4, and CXCR1. Opioid receptors have been shown to initiate the NF-κB pathway in cortical neurons, neuroblastoma cells, or the THP-1 macrophage-like cell line (40–42). While the effects of opioid receptor activation may vary with the cell type, NF-κB is clearly a critical component of opioid function (43).

Finally, activated GPCRs can initiate signaling pathways which lead to the stimulation of one or more of the members of the protein kinase C (PKC) family. There are at least 15 isoforms of this serine/threonine kinase family, and these enzymes are expressed by a wide variety of cell types in virtually all tissues. However, each of the PKC isozymes exhibit a unique set of tissue distribution patterns, subcellular localizations, and functions (44). These isoforms have been grouped into three PKC types, conventional (or classical) (PKCα, β, and γ), novel (PKCδ, ε, η, and θ), and atypical (PKCζ and PKCι/λ). The distinctions between these PKC isoforms is important because they can have significantly different activation requirements, and this in turn can have an impact on the nature of their enzymatic substrates. The conventional PKCs bind diacylglycerol (DAG), and this induces kinase catalytic activity and requires calcium, while the novel PKCs bind DAG but do not require calcium. Finally, the atypical PKCs do not bind DAG and do not require calcium, so the biochemical basis for enzymatic activation is distinct from the other PKC isoforms. However, all of the PKC members possess a highly conserved COOH-terminal catalytic domain, and an NH2-terminal regulatory region that contains a unique pseudosubstrate sequence that binds to the catalytic domain and maintains the enzyme in an inactive form (in the absence of the activating second messenger) (45).

There is accumulating evidence that the atypical PKCζ plays a critical role in the regulation of a number of metabolic processes. The primary PKCζ activation pathway is typically dependent on PI3K, which produces phosphatidylinositol PI-3,4,5-trisphosphate (PIP_3_) (46). PIP_3_ is free then to bind and activate phophoinositide-dependent protein kinase-1 (PDK1). The activation of PKCζ is dependent in part on the phosphorylation of Thr-410 within the activation loop, and following this step, autophosphorylation of PKCζ at Thr-560 (47). PKCζ also contains a Phox/Bim1 (PB1) domain near the NH2-terminus that mediates binding to protein scaffolds. Engagement of binding to the scaffold protein performs the same function as the binding of DAG to the C1 domain of conventional and novel PKCs (48). This disengages the pseudosubstrate, resulting in full PKC activity, and the atypical PKCs are typically constitutively active once bound to their scaffold protein (49). PKCζ plays an important role in the cross-regulation of both chemokine expression, and chemokine receptor function.

It is well-established that the PKCs participate in a number of functional activities of the opioid receptors. Moreover, activation of MOP by the highly selective synthetic MOP agonist [D-Ala^2^, *N*-Me-Phe^4^, Gly-ol^5^]enkephalin (DAMGO) results in translocation of PKC isoforms α, ε, and ζ to the plasma membrane, and these PKCs can participate in agonist-dependent MOP down-regulation (50). There is some evidence that KOP and MOP have distinct signaling patterns, and that this may be due to their ability to activate different sets of PKC isoforms and second messengers (51).

## Inflammatory Cell Expression of Opioid Peptides

It should not be surprising that opioid agonists are present at sites of inflammation, given the juxtaposition of cells producing both opioid-peptides and chemokines. Endogenous opioid peptides with agonist activities for MOP (β-endorphin, enkephalins), DOP (enkephalins, β-endorphin), and KOP (dynorphin) are produced by many inflammatory cells, including granulocytes, monocytes, macrophages, and lymphocytes (52, 53). On balance, these opioid peptides exhibit anti-inflammatory activity, which is in line with the capacity of opiate analgesics to inhibit inflammatory pain. For example, patients suffering with arthritis responded to morphine administration with decreased pain sensitivity, and a reduction in synovial inflammatory cells (54, 55).

Published evidence suggests that leukocytes release opioid peptides in response to a variety of stimuli. For example, leukocytes produce opioid peptides in response to *in vitro* stimulation with either corticotropic releasing factor (CRF), IL-1, or noradrenaline (56–60). Opioid peptide producing leukocytes have been reported to co-express chemokine receptors, formyl peptide receptors, and receptors for certain cytokines including IL-1 (59, 61–63). Granulocytes produce both β-endorphin and met-enkephalin in response to stimulation with CXCL2 or CXCL3, or mycobacteria-derived formyl peptide expression (63, 64). Recent evidence shows that alternatively activated macrophages (M2 macrophages) produce β-endorphin, dynorphin, and met-enkephalin when adoptively transferred to sites of inflammation *in vivo* (65). This result is in contrast to either classically activated macrophages (M1 macrophages) or non-polarized macrophages, which produce substantially lower levels of these opioid peptides. Similar results have been reported for T_H_ cells, which produce β-endorphin and met-enkephalin in inflamed tissue (66, 67).

In general, the opioid peptides exhibit anti-inflammatory activity, and there is evidence that these peptides contribute to wound healing. Evidence has been reported which show that opioid peptides exhibit mitogenic activity for epithelial cells, promote re-epithelialization and keratinocyte migration, and stimulate both cytokeratin and TGFβ (53, 68–71). In more advanced ischemic wounds, the local application of opioids promote wound closure, induce granulation tissue, stimulate epidermal and dermal organization, and up-regulate angiogenesis (72, 73). In contrast to these results, it should be pointed out other reports have suggested that opioid administration may slow wound healing (74, 75). The nature of the apparently opposing results in these studies remains uncertain.

Additional evidence that opioid peptides play a role in the inflammatory response *in vivo* has been provided by studies which show that inhibition of the extracellular degradation of opioid peptides leads to antinociception (76). In addition, MOP-knockout mice express increased levels of TNFα, IL-1β, IL-4, and IFNγ at sites of inflammation (77). Taken together, the results demonstrate that opioid peptides are produced at sites of inflammation, are produced by inflammatory cells, and appear to play an anti-inflammatory role in the immune response.

## Opioid-Mediated Regulation of Chemokine Expression

In general, opioids (particularly MOP agonists) mediate immunosuppressive activity at the level of cytokine expression. For example, the production of IFNγ, IL-2, IL-1β, TNFα are inhibited by MOP agonists (78–81). In contrast with these results, under the appropriate circumstances, MOP agonists may upregulate the expression of other pro-inflammatory cytokines. Peng et al. (82) have reported that both IL-12 and TNFα expression by murine peritoneal macrophages is elevated in response to morphine. Moreover, Roy et al. (83) have shown that morphine, at low doses, up-regulates the expression of both IL-6 and TNFα. These results establish that the MOP agonists can induce both pro- and anti-inflammatory activities.

The MOP-selective agonist DAMGO can upregulate CCL2, CXCL10, and CCL5 production by both non-activated and PHA-stimulated peripheral blood mononuclear cells (PBMCs) at both the mRNA and protein level (21). This effect is blocked by administration of the MOP-selective antagonist H-_D_-Phe –Cys-Tyr-_D_-Trp-Arg-Thr-Pen-Thr-NH_2_ (CTAP) indicating that this effect is mediated through MOP. In addition, Rock et al. (84) showed that morphine stimulates CCL2 production at both the mRNA and protein level in neurons, and this result was blocked by the addition of the MOP antagonist, β-funaltrexamine (β-FNA). Caco-2, an intestinal epithelial cell line, which was found to constitutively express MOP and KOP, and treatment with the selective MOP tetrapeptide, endomorphin-1 results in a significant increase in CXCL8 production (85, 86).

The biochemical basis for the induction of chemokine expression has been the subject of research reported from several laboratories. MOP agonists, including morphine, can up-regulate NF-κB activity in neuronal cells, including rat cerebral cortex neurons (40), and the NT2-N neuronal cell line (87). The activation of NF-κB has significant implications since it is critical for the expression of a large number of pro-inflammatory cytokines. Both morphine (83) and the synthetic MOP agonists endomorphin 1 and 2 (42) have been shown to induce NF-κB activity in monocyte/macrophage cell populations.

A more detailed examination of the biochemistry of MOP-induced CCL2 expression has shown that early, direct induction of CCL2 expression is dependent on the activation of NF-κB (19). These studies demonstrate that DAMGO treatment of human primary leukocytes results in significant up-regulation of CCL2 mRNA and protein by 4 h, and inhibition of NF-κB activation significantly reduces CCL2 expression. DAMGO administration induces NF-κB activation by 30 min, and this activation is dependent on the phosphorylation of the p65 subunit of NF-κB at Ser-311 (19). At this time the only kinase known to carry out this phosphorylation/activation step is PKCζ, and additional experiments demonstrated that the activation of MOP leads to the activation of PKCζ. Moreover, inhibition of PKCζ activity was found to inhibit the activation of NF-κB or the induction of CCL2. Finally, chromatin immunoprecipitation (ChIP) analysis demonstrated that MOP activation induced binding of NF-κB to the CCL2 promoter (19). It has been proposed that the induction of CCL2 following MOP activation is the result of a signaling pathway involving Gβγ, PI3K, PDK-1, and PKCζ (19). This signaling pathway may possibly form the biochemical basis for the MOP-induced expression of other cytokines and chemokines.

For other chemokines, the regulation of expression mediated by opioids may be indirect. This is true for CCL5, a chemokine which is induced only after 18–24 h following activation of MOP. There is some evidence that opioid-mediated modulation of cytokine expression is due to the intermediate induction of TGFβ (18). In this case, the inhibition of TNFα by morphine was found to require the initial induction of TGFβ. More recent studies were carried out to determine whether the MOP-induced expression of CCL5 might require up-regulation of TGFβ. In experiments with primary human monocytes, the activation of MOP resulted in a significant induction of TGFβ by 8 h, and the up-regulation of CCL5 was blocked in the presence of anti-TGFβ neutralizing antibody (20). On the other hand, the MOP-induced up-regulation of both CCL2 or CXCL10 was found to be insensitive to anti-TGFβ antibody treatment. It should be pointed out that TGFβ has been reported to directly induce CCL2 expression through the activation of ERK and p38 MAPK pathways in mesangial cells (88). Moreover, TGFβ has been reported to up-regulate CCL2 in murine osteoblasts via the transcription factor AP-1 (89, 90).

While most of the work in this area has been done with MOP, there is evidence that KOP can regulate the expression of certain chemokines. Using the κ-opioid selective agonist U50,488H (trans-3,4-dichloro-N-methyl-N[2-(1-pyrolidinyl)cyclohexyl]benzeneacetamide methanesulfonate) studies have shown that activation of KOP may down-regulate CCL2 production in primary human astrocytes stimulated with the HIV-1 nuclear protein, Tat (91). In addition, the activation of KOP also inhibits the expression of CXCL8 in the presence of IL-1β (86). These results are in line with other work which suggests that KOP generally exerts immunosuppressive activity (13). For example, the activation of KOP results in a reduction in the expression of IL-1β, IL-6, and TNFα in primary macrophages and macrophage/monocyte cell lines (92).

## Opioid-Mediated Regulation of Chemokine Receptor Expression

In general, the activation of MOP leads to the up-regulation of several chemokine receptors. Morphine administration induces the expression of CCR3, CCR5, and CXCR2 in the U87 human astrocytoma cell line (93). The astrocytoma/glioblastoma cell line also up-regulates CCR3 and CCR5 expression following morphine treatment. Studies carried out with the more MOP-selective agonist DAMGO have shown that activation of MOP induces a substantial increase in the expression of both CCR5 and CXCR4 (22). In contrast, activation of KOP induces a significant down-regulation of CXCR4 (23). These studies showed that the reduced CXCR4 expression leads to a significant reduction in the susceptibility of T cells to HIV infection (23, 94). These results are in contrast to results showing that KOP activation increases expression of CCR2 by developing immature murine T cells (95). These results are indicative of the contrasting effects of opioids on chemokine receptor expression and function.

In an effort to understand the mechanisms involved in the regulation of chemokine receptor expression, studies were conducted with human primary leukocytes which showed that the up-regulation of CXCR4 was dependent on the initial expression of TGFβ (20). In a manner similar to the studies reviewed above on CCL5, the results showed that treatment with neutralizing anti-TGFβ blocked the MOP-induced expression of CXCR4. In addition, these results also showed that both monocytes and T cells exhibit increased CXCR4 following either treatment with TGFβ, or the MOP agonist DAMGO (20). Interestingly, the MOP-induced increase in CCR5 expression was not dependent on TGFβ production, and TGFβ does not induce CCR5 up-regulation.

It should be acknowledged that other cytokines (in addition to TGFβ) may also regulate the opioid-induced modulation of chemokine receptor expression. For example, results have been reported which show that TNFα induces the expression of CCR5 (96, 97). Moreover, IFNγ is able to up-regulate the expression of CCR5 in monocytes (98), and MOP activation can induce an up regulation of both TNFα and IFNγ [reviewed in (14)]. The levels of expression of these and additional cytokines may determine whether a given opioid receptor yields a pro- or anti-inflammatory effect in physiological conditions.

Additional work to understand the biochemical basis for opioid-regulation of chemokine receptor expression has been carried out for KOP. Using both human peripheral blood leukocytes, and the human microglial cell line CHME-3, studies have demonstrated that KOP-induced down-regulation of CXCR4 is dependent on JAK2, STAT3, and IRF2 (23). These studies showed that the activation of KOP resulted in the activation of several transcription factors, including the STAT proteins 1, 3–6. KOP activation also resulted in the activation of IRFs. More detailed analysis showed that KOP mediated significant activation of both JAK2 and STAT3 by 5 min following KOP activation, and both JAK2 and STAT3 inhibitors blocked the down-regulation of CXCR4 mediated by KOP (23). In addition, KOP activation induced the expression of both IRF1 and IRF2, and the induction of both were blocked by JAK2 and STAT3 inhibitors. CHME-3 cells which expressed inhibitory siRNA for IRF2 failed to exhibit KOP-mediated inhibition of CXCR4 expression, and ChIP analysis demonstrated that KOP activation resulted in substantial binding of IRF2 to the CXCR4 promoter. These studies suggest that a KOP activation initiates a signaling pathway which is composed of KOP–JAK2–STAT3–IRF2–CXCR4. Taken together, these results were surprising since this is the first evidence that KOR activates the JAK/STAT signaling pathways. On the other hand, there is evidence that MOP and DOP are able to induce the phosphorylation of STAT3 and STAT5 (99–102). The JAK/STAT signaling mediators are well-known to play a critical role in the functions of receptors for a number of cytokines, including IL-2, 3, 4, 6, 12, 13, IFNα/β, IFNγ, and GM-CSF. On the other hand, there is limited evidence that these proteins play a significant role in the function of the chemokine receptors. There is evidence that JAK2 and JAK3 may contribute to the chemotaxis activity of CXCR4 (103, 104). In addition, the chemotaxis activity of CCR7 has been reported to be JAK3-dependent (105). These studies remain controversial, and Moriguchi et al. (106) have reported studies which suggest that CXCR4 function is independent of JAK2 and JAK3.

## Opioid Receptor-Induced Desensitization of Chemokine Receptor Functional Activity

The regulation of GPCR functional activity takes place at multiple levels, including controls of the expression of either the receptor or the binding ligand, traffic of the receptor to or from the outer membrane, and expression/positioning of scaffold proteins which associate with the receptor. However, the process of desensitization of the receptor is a critical aspect of the regulation of receptor function, and this process is essential in order to maintain the normal functioning of cells. Desensitization also works to prevent excessive signaling through the receptor, and allow the cell to utilize the receptor for discrete periods of time.

In the first seconds following agonist binding to the GPCR, G proteins are uncoupled, and downstream signaling pathways are initiated (107). The response reaches a peak within seconds, and rapidly declines while the agonist-receptor complex remains intact. As the signaling capacity slows, the process of homologous desensitization is initiated, and this process can bring the signaling to an end. Responses of the receptor to re-stimulation are reduced, and the receptor may remain on the outer membrane (but in an unresponsive state), or more commonly, the receptor may be internalized. Homologous desensitization, in which the receptor is desensitized following receptor activation by the homologous agonist, typically occurs as a result of receptor phosphorylation by one or more G protein-coupled receptor kinases (GRKs). The GRKs are serine/threonine kinases, there are seven GRK subtypes, and the expression patterns for the GRKs vary among cell types (108). Following phosphorylation, β-arrestin may be recruited to the receptor, and this complex can promote the processes which are required for internalization. Once internalized, the desensitized receptor can be degraded or re-sensitized, and in some circumstances, the sensitized receptor may be returned to the outer membrane.

An alternative process for GPCR desensitization involves a process in which one GPCR is activated, and initiates a signaling process which leads to the desensitization of a second, unrelated, and non-ligated receptor. This process is referred to as heterologous desensitization (or cross-desensitization), and in most cases is dependent on the activation of second-messenger-dependent kinases. For example, the activation of G_s_ coupled receptors typically activates adenyl cyclase, resulting in the formation of cyclic AMP, and the activation of PKA. The G_i/o_ coupled receptors typically result in the activation of PKC isoforms, and PKA or PKC mediates phosphorylation of serine/threonine residues in the carboxy-terminal tail of the GPCR. The serine and/or threonine phosphorylation sites are distinct for PKA, PKC, and GRK enzymes, and the location and/or number of these sites may determine the susceptibility of a given GPCR to heterologous desensitization (109). As with the process of homologous desensitization, receptors that undergo heterologous desensitization may not be internalized, but remain on the cell surface in a refractory state (107). It is not clear whether receptors that have undergone heterologous desensitization associate with β-arrestin, and little is known about the internalization biochemistry for these receptors. Nevertheless, internalization is typically not required in order for the receptor to be dephosphorylated and re-sensitized (110–112).

Early work on heterologous desensitization showed that activation of the formyl peptide receptor (FPR) induced heterologous desensitization of the C5a receptor (C5aR) and CXCR1, but not platelet-activating factor receptor (PAFR) or the leukotriene B receptor (LTBR) (113). On the other hand, the activation of either PAFR or LTBR were able to cross-desensitize FPR. These results demonstrate the existence of a hierarchy among GPCRs in terms of susceptibility to heterologous desensitization. For example, the strength of the receptors to induce heterologous desensitization is approximately FPR > C5aR > CXCR1, while the susceptibility to cross-desensitization was reversed and approximately CXCR1 > C5aR > FPR (113).

The first report of opioid-induced heterologous desensitization of chemokine receptors was reported by Grimm et al. (114), in which met-enkephalin and morphine administration inhibited the activity of CXCR1, CXCR2, CCR1, CCR5, or CCR2. In contrast, the opioid receptor activation had no effect on the function of FPR expressed by either neutrophils or monocytes. Additional studies also showed that the both MOP and DOP could mediate this cross-desensitization, and as expected, the biochemistry of these processes involved target receptor phosphorylation (114, 115). Additional studies have shown that MOP-induced heterologous desensitization of CCR5 is associated with a loss of HIV co-receptor function, but the MOP-induced cross-desensitized CCR5 is not internalized (116). In contrast, neither MOP or DOP induce cross-desensitization of CXCR4 in either T cells or monocytes, and in this case the susceptibility to X4-tropic HIV is not altered by the opioid receptor activation.

**Figure 1:**
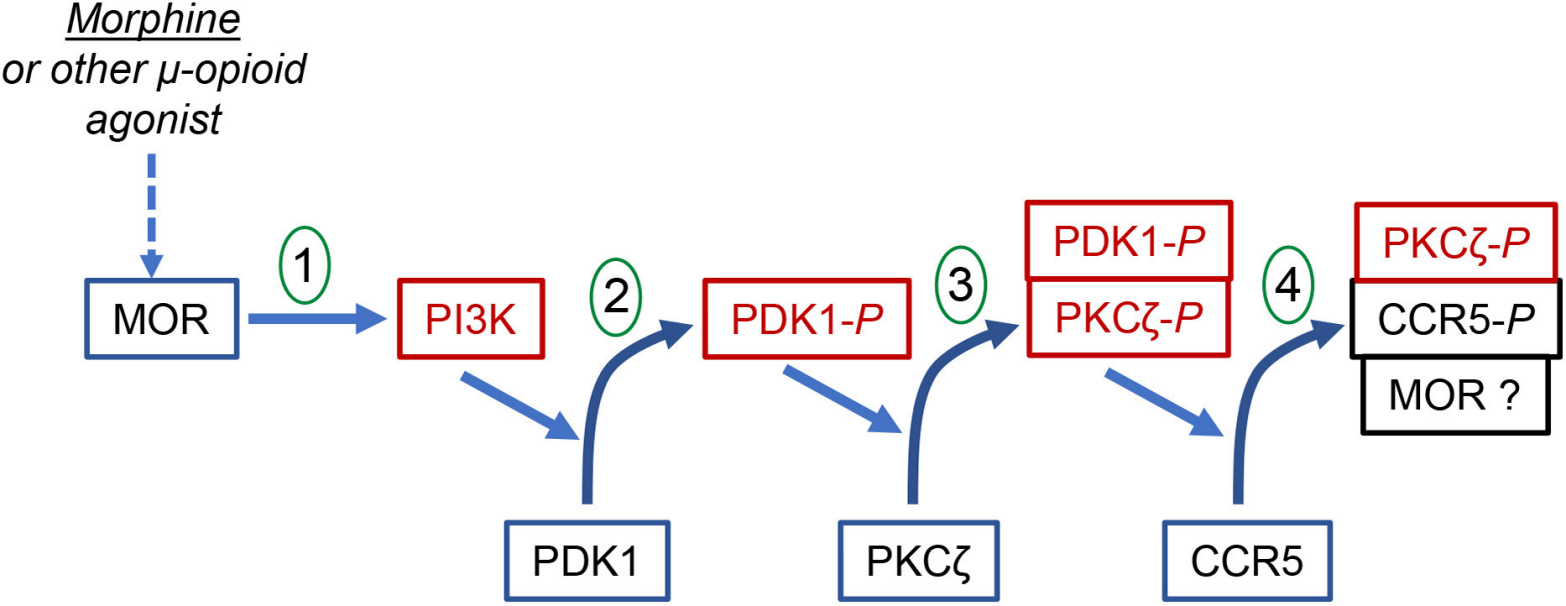
The signal transduction process for the MOR-induced heterologous desensitization of CCR5. The diagram shows the step-wise phosphorylation steps leading to the phosphorylation and desensitization of CCR5. Step 1 shows the activation of MOR leading to the activation of PI3K. Step 2 shows the PI3K-dependent activation and phosphorylation of PDK1. Step 3 shows the PDK1-dependent phosphorylation and activation of PKCζ. This step appears to involve the formation of PDK1-PKCζ heterodimers. Step 4 shows the PKCζ-dependent phosphorylation and desensitization of CCR5. This step involves the formation of a complex of phorphorylated PKCζ and phosphorylated CCR5. It is possible that a larger complex which includes MOR may also be formed.

It has been suggested that the selective nature of heterologous desensitization is due to the activation of a limited subset of PKC isozymes (117), and that the ability of these to phosphorylate a potential target receptor would determine the level of susceptibility. Zhang et al. (117) showed that activation of MOP by either morphine or met-enkephalin activates PKC isoforms δ, η, μ, and ζ in monocytes, and the calcium-dependent classical PKCs are not activated. The failure to activate the classical PKCs may indicate a limited range of target receptors for MOP-induced cross-desensitization. Additional studies reported by Song et al. (118) using either a selective pseudosubstrate inhibitor peptide, inhibitor siRNA expression, or expression of a dominant-negative mutant, showed that cross-desensitized CCR5 is phosphorylated by PKCζ. These investigators went on to show that the MOP-mediated induction of PKCζ is dependent on the initial activation of PDK1, and using fluorescent resonance energy transfer techniques and co-immunoprecipitation, the activation of PKCζ and desensitization of CCR5 occurred as a result of the formation of a PDK1-PKCζ-CCR5 complex. These results are consistent with the report of Chen et al. (119) who demonstrated MOP-CCR5 complex formation, and suggested that the complex formation may contribute to the heterologous desensitization. A diagram showing the signaling pathway for the MOP-induced cross-desensitization of CCR5 is presented in Figure 1.

The selective activation of PKC isoforms is an inherent property of many members of the GPCR superfamily. In addition, the capacity of a given GPCR to utilize one or more PKC isozymes to mediate heterologous desensitization is an important aspect of the hierarchy of GPCR strength of cross-desensitization. For example, studies of the interactions between CXCR1, CXCR2 and CCR5 show that CXCR1 and CCR5 exhibit bi-directional heterologous desensitization (120). The activation of CXCR1 and CCR5 induce strong and sustained PKCε, but weak and transient PKCα, βI and βII. Inhibition of PKCε, but not the other PKC isozymes, blocked this heterologous desensitization. Moreover, CXCR2 failed to induce activation of PKCε, and failed to desensitize either CXCR1 or CCR5. These results demonstrate that both PKCε (when activated by CXCR1) or PKCζ (when activated by MOP) can desensitize CCR5.

The finding of MOP-CCR5 heterodimerization offers an interesting level of potential complexity in the cross-talk between opioid and chemokine receptors. It has been reported that for some GPCR heterodimers, the activation of one dimer partner leads to an inhibition in the function of the other partner (121). For example, in studies of the α_2A_-adrenoceptor-MOP receptor heteromer, morphine binding inhibited α_2A_-adrenoceptor signaling (122). Moreover, this has been attributed to a morphine-stimulated alteration in the conformation of the α_2A_-adrenoceptor, based on results from dynamic intramolecular FRET (123). In fact, the change in α_2A_-adrenoceptor conformation was found to occur within 0.4 s of the activation of MOP, which is more rapid than the rate of G protein activation. It is possible that for the interaction between MOP and CCR5 there may be additional negative effects on CCR5 function mediated by MOP activation that occur at the level of CCR5 conformation.

As noted above, the activation of either MOP or DOP do not induce heterologous desensitization of CXCR4. However, it is apparent that both KOP and NOP are able to mediate cross-desensitization of this chemokine receptor. NOP is abundantly expressed by hematopoietic cells, including T cells. Studies with human peripheral blood T cells and monocytes shows that CXCR4 fails to mediate a chemotaxis response to CXCL12 following the activation of NOP (124). The desensitization of CXCR4 occurred in the absence of detectable internalization of this receptor, in contrast to homologous desensitized CXCR4 in which there was substantial internalization. Furthermore, the NOP-induced heterologous desensitization of CXCR4 also resulted in a loss of CXCR4 HIV co-receptor function. In addition, results have been reported which show that CXCR4 and KOP are able to carry out bi-directional heterologous desensitization (125). The CXCR4-induced cross-desensitization of KOP was not associated with significant internalization, but the KOP-induced desensitization of CXCR4 was associated with partial CXCR4 internalization. Nevertheless, it appears that opioid receptor-mediated cross-desensitization of these chemokine receptors does not require a high level receptor internalization, and the mechanism of desensitization is independent of internalization.

It is important to point out that sustained stimulation of MOP in neurons results in the inhibition of CXCR4 functional activity (126, 127). These studies showed that the MOP-induced desensitization of CXCR4 required the upregulation of ferritin heavy chain (FHC) (127, 128). FHC has been shown to negatively regulate CXCR4 signaling activity in both neuronal and non-neuronal cells, and FHC and CXCR4 co-immunoprecipitates after stimulation with morphine (129, 130). This binding interaction appears to inhibit coupling of CXCR4 to G proteins, and in this way interfere with the signaling activity of the receptor (131). This mechanism of opioid-mediated regulation of CXCR4 may have significant implications for the regulation of the immune response. Additional work on the role of FHC in immune function will be necessary to address this issue.

## Chemokine-Induced Desensitization of Opioid Receptors

Pain is one of the cardinal signs of inflammation, and the mechanisms that are responsible for the increase in sensitivity to pain are complex. Elevated levels of pro-inflammatory chemokines have been measured at sites of chronic inflammation, and these chemokines contribute to the increase in pain sensitivity (132). Based on the knowledge of cross-talk between chemokine and opioid receptors, studies have been conducted to determine the capacity of various pro-inflammatory chemokines to induce heterologous desensitization of opioid receptors. Results have been reported which demonstrate that the activation of CCR2, CCR5, CCR7, or CXCR4 rapidly induces cross-desensitization of both MOP and DOP in primary human monocytes and T cells (115, 133). Additional studies have shown that the activation of CCR1, CCR2, and CXCR1 induces the cross-desensitization of MOP in both neuronal and non-neuronal cells (134).

In an effort to examine the physiological consequences of the chemokine-opioid receptor cross-talk, experiments were conducted by administration of chemokines into the periaqueductal gray matter (PAG) of the brain, and then the ability of MOP to elicit an analgesic response was determined. Stimulation of the MOP in the PAG is known to elicit an analgesic response (a depressed sensation of pain). In these studies the pretreatment of the PAG with agonists for either CCR5 or CXCR4 inhibited the ability of MOP to mediate a normal analgesic response to a MOP agonist (133). More recent work has demonstrated that the activation of CCR1, CCR5 and CXCR4 in the PAG result in cross-desensitization of both MOP and DOP (135). Results reported by Chen et al. (136) have shown that the activation of CX3CR1 in the brain induces cross-desensitization of MOP, DOP, and KOP. Finally, as mentioned above, a study reported by Finley et al. (125) shows that activation of CXCR4 results in cross-desensitization of KOP (125). Taken together, these studies suggest an extensive degree of regulation of the function of opioid receptors that is mediated by chemokine receptors. A very recent report shows that the co-administration of chemokine receptor antagonists potentiates the capacity of morphine to generate an analgesic response in a model of inflammatory pain (132). These results suggest that the results on heterologous desensitization can be clinically translated by interfering with the chemokine-mediated desensitization of MOP function in conditions involving inflammatory pain. The capacity of certain cytokines and chemokines to alter the perception of pain has recently been reviewed, including the cross-desensitization of opioid receptors following activation of chemokine receptors (137, 138).

## Conclusions

It is worth noting that there are several prominent physiological consequences of the heterologous desensitization between opioid and chemokine receptors. It should be pointed out that most of the work reviewed above was conducted by assessing the effects following acute administration of opioids and/or chemokine agonists. It is conceivable that chronic agonist administration may yield results which are qualitatively or quantitatively distinct from those described herein. The consequences of chemokine-induced desensitization of opioid receptors during conditions of inflammatory pain were discussed above. On the other hand, the consequences of selective cross-desensitization of chemokine receptors should be discussed. For example, the inflammatory response is dependent on the appropriate guidance of inflammatory cells to the site of inflammation. The recruitment of these inflammatory cells first requires that the cells orient the expression of chemoattractant receptors, and the underlying cytoskeletal matrix (polarization), to prepare the cell for the journey to the inflammatory site. However, this guidance must occur in the presence of a mixture of a large number of chemoattractants. There must be hierarchies of these chemoattractant stimuli that allow for a refined directional signal. It has been pointed out that receptor desensitization plays an important part in separating the influence of the “strong” chemoattractant signals (“end point signals”), and potentially overcome the influences of less dominant chemokines (“intermediate chemokines”) (139). It is suggested here that heterologous desensitization is an important part of this guidance process, since its influence is both rapid and selective. For example, the high-affinity formyl peptide receptor, FPR1, plays a critical role in the initial recruitment of neutrophils to sites of inflammation and/or infection. The recruitment of these cells by this receptor is essential for early resistance to a number of infectious agents, and the formyl peptide agonists are produced by endogenous cellular damage, as well as by infectious agents. In other words, the source of agonist for FPR1 is present at an early stage of the inflammatory process. However, other chemoattrants are also present in the tissue, and the FPR1-mediated cross-desensitization of the interfering chemokine receptors would protect the function of FPR1. Studies reported by Bednar et al. (109) show that FPR1 successfully mediates cross-desensitization of CCR1, but not CCR2. The desensitization of CCR1 is dependent on PKCβ, and this appears to explain the inability of FPR1 to desensitize CCR2. Bednar et al. (109) proposed that FPR1 uses heterologous desensitization of CCR1 (and likely other receptors) to exert “traffic control” by rapidly inhibiting the interference by “low-priority” chemoattractants, without impeding the influence of “high-priority” CCR2-mediated chemoattraction.

The contribution of heterologous desensitization to the inflammatory response is only partially understood. The capacity of one GPCR to inhibit the activity of another GPCR is likely to play a role in the regulation of a number of receptor functions. Studies reported thus far deal with receptor responses, such as chemotaxis and calcium flux reactions, and these are important aspects of inflammatory cell functions. However, GPCRs also play a role in a number of other functions, including cell growth and survival, the production of pro-and anti-inflammatory mediators, the activation of adhesion molecules, and the susceptibility to infection by a number of infectious agents. We are still in the process of learning the spectrum of GPCR functions that are regulated by heterologous desensitization.

## Author Contributions

TR reviewed the literature and prepared the manuscript.

### Conflict of Interest

The author declares that the research was conducted in the absence of any commercial or financial relationships that could be construed as a potential conflict of interest.
